# Novel *PAX9* compound heterozygous variants in a Chinese family with non-syndromic oligodontia and genotype-phenotype analysis of *PAX9* variants

**DOI:** 10.1590/1678-7757-2022-0403

**Published:** 2023-03-27

**Authors:** REN Jiabao, Ya ZHAO, Yunyun YUAN, Jing ZHANG, Yulin DING, LI Meikang, AN Yilin, CHEN Wenjing, Li ZHANG, LIU Boyu, Shushen ZHENG, Wenjing SHEN

**Affiliations:** 1 Hebei Clinical Research Center for Oral Diseases School and Hospital of Stomatology Hebei Medical University Shijiazhuang PR China Department of Prosthodontics, Hebei Key Laboratory of Stomatology, Hebei Clinical Research Center for Oral Diseases, School and Hospital of Stomatology, Hebei Medical University, Shijiazhuang 050017, PR China.; 2 Hebei Clinical Research Center for Oral Diseases School and Hospital of Stomatology Hebei Medical University Shijiazhuang PR China Department of Orthodontics, Hebei Key Laboratory of Stomatology, Hebei Clinical Research Center for Oral Diseases, School and Hospital of Stomatology, Hebei Medical University, Shijiazhuang 050017, PR China.; 3 Xingtai Medical College Xingtai Hebei China Xingtai Medical College, Xingtai 054000, Hebei, China.; 4 Hebei Clinical Research Center for Oral Diseases School and Hospital of Stomatology Hebei Medical University Shijiazhuang PR China Hebei Key Laboratory of Stomatology, Hebei Clinical Research Center for Oral Diseases, School and Hospital of Stomatology, Hebei Medical University, Shijiazhuang 050017, PR China.

**Keywords:** Tooth agenesis, Non-syndromic oligodontia, Paired Box 9 Protein, Whole-exome sequencing, Genotype-phenotype

## Abstract

Studies have reported that >91.9% of non-syndromic tooth agenesis cases are caused by seven pathogenic genes.

**Objective:**

To report novel heterozygous PAX9 variants in a Chinese family with non-syndromic oligodontia and summarize the reported genotype-phenotype relationship of PAX9 variants.

**Methodology:**

We recruited 28 patients with non-syndromic oligodontia who were admitted to the Hospital of Stomatology Hebei Medical University (China) from 2018 to 2021. Peripheral blood was collected from the probands and their core family members for whole-exome sequencing (WES) and variants were verified by Sanger sequencing. Bioinformatics tools were used to predict the pathogenicity of the variants. SWISS-MODEL homology modeling was used to analyze the three-dimensional structural changes of variant proteins. We also analyzed the genotype-phenotype relationships of PAX9 variants.

**Results:**

We identified novel compound heterozygous *PAX9* variants (reference sequence NM_001372076.1) in a Chinese family with non-syndromic oligodontia: a new missense variant c.1010C>A (p.T337K) in exon 4 and a new frameshift variant c.330_331insGT (p.D113Afs*9) in exon 2, which was identified as the pathogenic variant in this family. This discovery expands the known variant spectrum of *PAX9* ; then, we summarized the phenotypes of non-syndromic oligodontia with *PAX9* variants.

**Conclusion:**

We found that *PAX9* variants commonly lead to loss of the second molars.

## Introduction

Hypodontia, oligodontia, and anodontia are forms of selective tooth agenesis, which refers to the reduction in the number of teeth caused by gene variants or environmental interference during tooth development.^1 , 2^This condition can be further subdivided into non-syndromic tooth agenesis and syndromic tooth agenesis according to the presence or absence of developmental abnormalities of other organs and systems.^1^ Non-syndromic oligodontia (NSO) refers to the congenital absence of six or more permanent teeth (excluding the third molar) without abnormal development of other organs. The incidence of NSO varies from 0.1% to 0.5% depending on race and region.^2^

Dental organogenesis involves a series of complex epithelial-mesenchymal interactions,^3^ involving more than 200 genes^4 , 5^ and predominantly the TGF-β/BMP, Wnt/β-catenin, Eda/Edar/NF-κB, and SHH signaling pathways.^6^ *PAX9* , *AXIN2* , *EDA* , *LRP6* , *MSX1* , *WNT10A* , and *WNT10B* have been identified as the most common genes responsible for non-syndromic tooth agenesis. *EDAR* , *EDARADD* , *KRT17* , *NEMO* , and *KDF1* are also associated with non-syndromic tooth agenesis.^7 , 8^

*PAX9* is a member of the paired box (PAX) family of transcription factors, which play key regulatory roles in embryonic development and organogenesis. The gene is located on chromosome 14q13.3, consists of four exons (NM_001372076.1), and encodes a protein composed of 341 amino acids. The protein contains a paired-domain (PD),^9^ which consists of two structurally different helix-turn-helix motifs (the N-terminal subdomain and the C-terminal subdomain),^10^ and an octapeptide motif (OP) of unknown function. Mouse model studies have shown that the transcription factor *PAX9* is expressed in the dental mesenchyme during the initial stages of tooth development and is critical for the transfer of odontogenic potential from the odontogenic epithelium to the dental mesenchyme.^11^

In this study, we screened 28 NSO families by whole exon sequencing (WES) and identified and characterized the novel *PAX9* compound heterozygous variants in a Chinese family with non-syndromic oligodontia. Furthermore, we summarized the reported genotypes and phenotypes of *PAX9* variants to provide a theoretical basis for inferring genotypes from clinical phenotypes.

## Methodology

### Subjects

A cohort of 28 unrelated patients with NSO (average age 23.7 years old; 16 females and 12 males) was recruited in this study by referral from the Department of Prosthodontics in Hebei Medical University Hospital of Stomatology (China) during the period from 2018 to 2021. These patients confirmed that their missing permanent teeth were not due to extraction or injury. Phenotypic characterization of all patients included intraoral examination and panoramic radiographs to verify the number and pattern of missing teeth. In addition, 100 healthy volunteers were used as control. The inclusion criteria for healthy conditions (control) were: adults (22-55 years old) with a complete permanent dentition (28 teeth without third molars or 28-32 teeth, including third molars), without extra teeth or congenital tooth deficiency. They had a healthy physical condition, without organ or system diseases. This study was approved by the Ethics Committee of the School and Hospital of Stomatology, Hebei Medical University (NO: [2016] 004) and written informed consent was obtained from all patients.

### Peripheral blood sample collection and DNA extraction

Peripheral venous blood samples (2 ml) were collected from the probands, their available family members, and 100 unrelated healthy control volunteers. Genomic DNA was extracted using a blood genomic DNA extraction kit [Beijing Tiangen Biochemical Technology] following the manufacturer’s instructions and, then, stored at -20^0^C for future use.

### Whole-exome sequencing, Sanger sequencing and pathogenicity prediction

The genomic DNA of the proband was sent for WES sequencing by iGeneTech (Beijing, China). This process involved the establishment of a DNA library, and sequencing of the exons of the target region using the Nova6000 platform (Illumina. Inc., USA) after quality inspection and quantification. Sequencing yielded more than 25,600 Mb original bases; the sample reached an average target depth of 137×, exceeding 99.8% coverage. Clean readings from each sample were aligned with the human reference genome sequence (GRCh37/HG19) using Burrows-Wheeler Aligner (BWA V0.7.15). Single nucleotide polymorphisms (SNPs) and insertions and deletions (indels) were identified by SAMtools and the genome analysis tool GATK V3.7, and then annotated by ANNOVAR to determine the genetic information, functional information, possible detrimental effects, and so on corresponding to the variant site.

Candidate variants were identified according to the following criteria: (1) Known pathogenic genes; (2) Minor Allele Frequency (MAF) <0.01 in ExAC or 1000 genomic data; (3) Predicted to be pathogenic by Sorting Intolerant from Tolerant (SIFT), PolyPhen-2 or MutationTaster. Bidirectional primers of *PAX9* gene containing the predicted pathogenic *loci* were designed and verified by Sanger sequencing and TA cloning sequencing. The reference sequence of *PAX9* is NM_001372076.1.

### Conservation and structural modeling of the *PAX9* variants

For conservation analysis, the amino acid sequences of *PAX9* in six different species human (>NP_006185.1), cattle (>NP 001179298.1), chicken (>NP 990243.3), dog (>XP 03852 9399.1), house mouse (>NP 035171.1), and rhesus monkey (>NP 001035507.2) were obtained from the UniProtKB database (https://www.ncbi.nlm.nih.gov/). Clustal Omega (https://www.ebi.ac.uk/Tools/msa/clustalo/) was used to conduct the multiple sequence alignment and sequence logos were performed with WebLogo V2.8.2 (http://weblogo.berkeley.edu/).

For tertiary structural analysis, the *PAX9* protein structure was obtained from the Protein Data Bank (http://www.rcsb.org/). PyMol v2.1 (Molecular Graphics System, DeLano Scientific, CA, USA) was used to visualize the three-dimensional structure and analyze the structural changes.

### Genotype-phenotype analysis

A literature review was performed through searching PubMed from 1993 to 2022 using the search terms “ *PAX9* variants” or “ *PAX9* mutations”. Reports without detailed phenotype information were discarded. Finally, phenotype data of 157 non-syndromic tooth agenesis patients from articles plus the three patients in the present study were gathered for genotype-phenotype analysis. The phenotype composition of the 160 patients was analyzed. We found that *PAX9* variants mainly correlated with NSO. Therefore, the missing pattern of 132 patients of NSO was further characterized. The number and rate of missing teeth were estimated.

## Results

### Pedigree analysis and clinical findings

**Figure 1:**
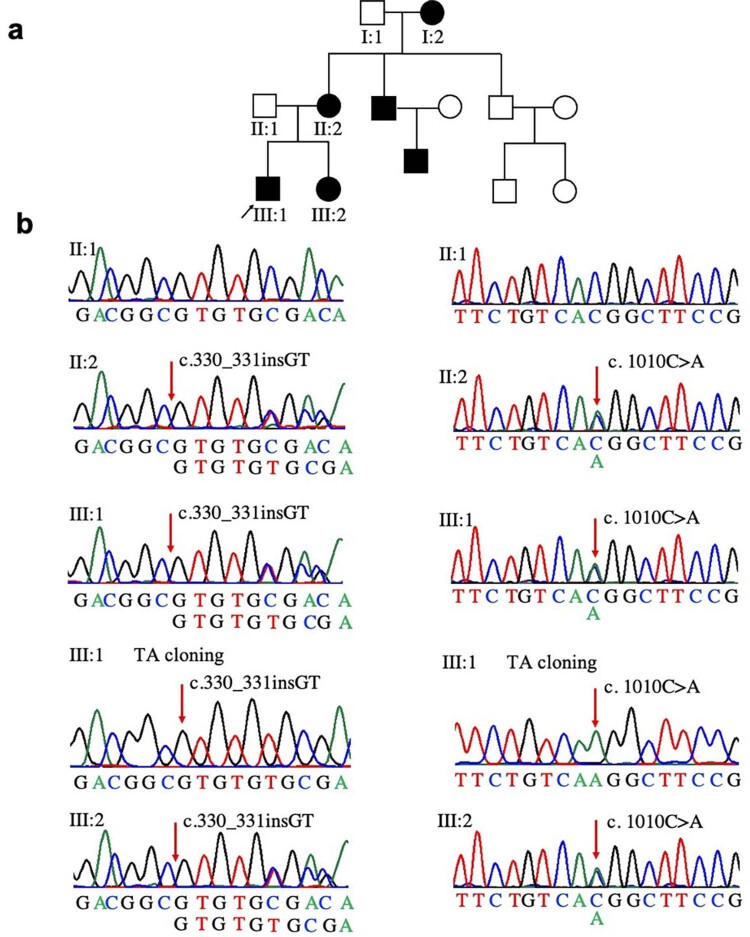
Identification of a compound heterozygous *PAX9* variant in a Chinese family with non-syndromic oligodontia. (a) The pedigree of the Chinese family. The black arrow indicates the proband. (b) DNA sequencing chromatograms of the family and TA cloning sequencing of the proband showing a new frameshift variant c.330_331insGT (p.D113Afs*9) in exon 2 and a new missense variant c.1010C>A (p.T337K) in exon 4 (reference sequence NM_001372076.1)

Pedigree analysis was constructed ( Figure 1a ) based on family histories provided by the proband’s mother and maternal grandmother. The proband and his family members had no signs of syndromes, no birth defects, and no ectodermal abnormalities correlating with facial appearance, hair, skin, nails, or sweat glands.

**Figure 2:**
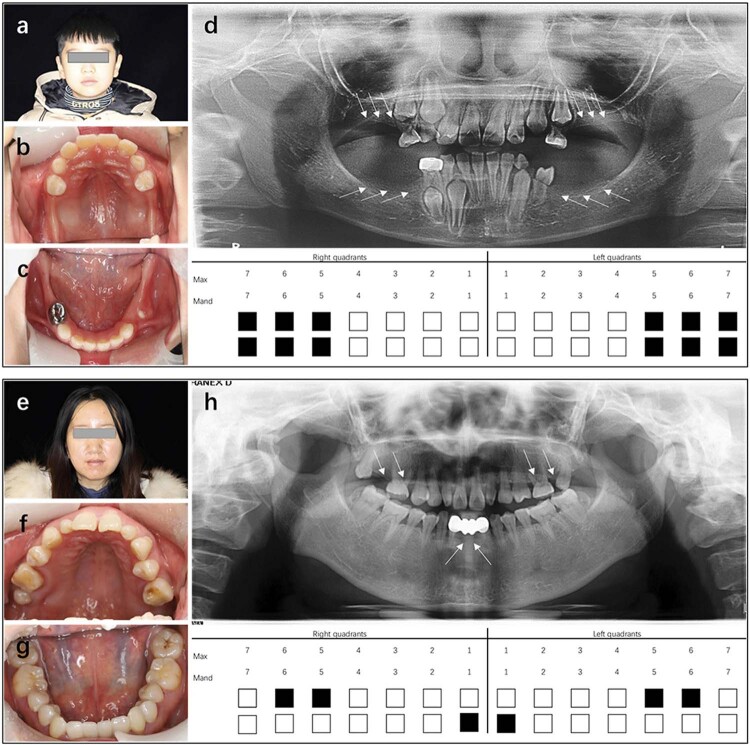
Dental characteristics of a Chinese family with non-syndromic oligodontia. a-d: (a) Facial characteristics of the proband; (b-c) Intraoral images; (d) Panoramic radiographs and schematic diagram of missing teeth; (e) Facial characteristics of the proband’s mother; (f-g) Intraoral images; (h) Panoramic radiographs and schematic of missing teeth. Black squares indicate missing teeth; Max, maxillary; Mand, mandibular

The proband ( Figure 2 a-d ) was a 9-year-old Chinese Han male who was diagnosed with NSO based on the examination results. The proband (III:1) had congenitally loss of 12 permanent teeth (excluding third molars) and five deciduous teeth (55, 65, 74, 75, and 85); The proband’s mother (II:2) had congenital loss of six permanent teeth ( Figure 2 e-h ). The proband’s younger sister had congenital loss of four deciduous teeth (all second deciduous molars), and 11 permanent teeth (Supplementary Figure). The proband’s grandmother (I:2) and maternal uncle and cousin were also affected by congenital tooth agenesis; however, their medical records were not accessible to verify their tooth phenotype.

### A novel compound heterozygous *PAX9* variant

A novel compound heterozygous variant of *PAX9* was found in this family consisting of a new frameshift variant c.330_331insGT (p.D113Afs*9) in exon 2 and a new missense variant c.1010C>A (p.T337K) in exon 4 ( Fig.1b ). Both the proband and his sister had *PAX9* variants inherited from their mother in an autosomal-dominant inheritance pattern. In addition, these two variants were not found in the 100 healthy controls, ExAC nor 1000G. The c.330_331insGT (p.D113Afs*9) variant resulted in termination of *PAX9* protein translation at position 121, whereas the c.1010C>A (p.T337K) variant resulted in an amino acid at position 337 that is not present in the truncation. Therefore, c.330_331insGT (p.D113Afs*9) was predicted to be the main pathogenic locus in the family.

### Bioinformatics analyses and structural modeling

**Figure 3:**
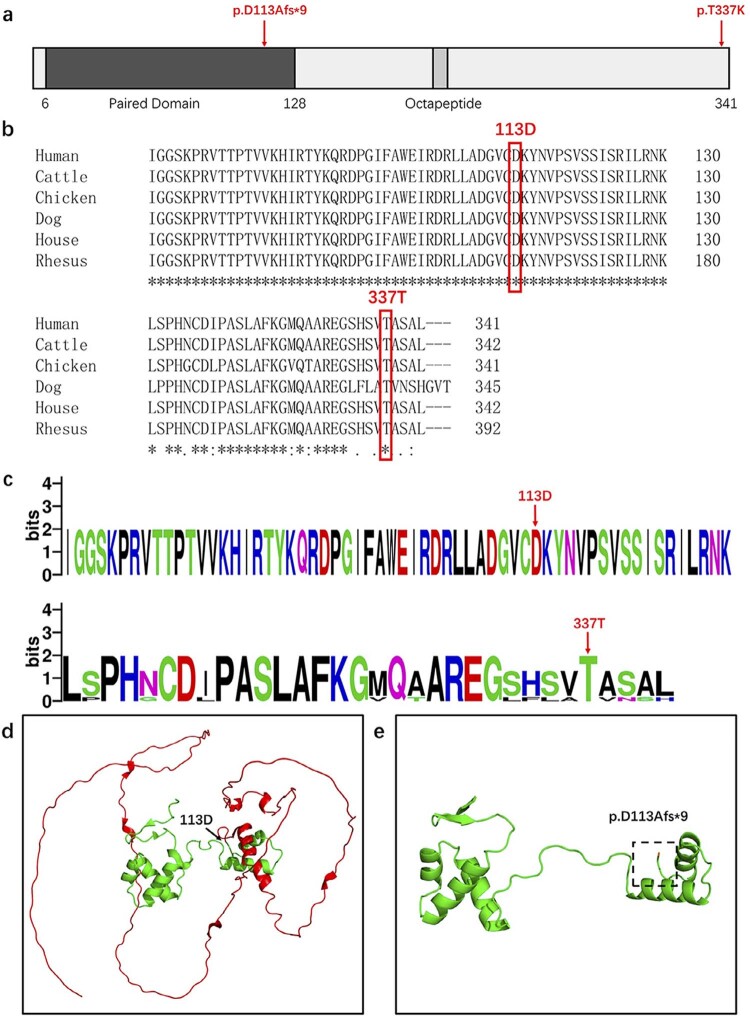
Conservation and bioinformatics analysis and structural modeling of *PAX9* . (a) Schematic diagram of the wild-type *PAX9* protein and the localization of the novel compound heterozygous *PAX9* variant identified in this study. (b) Conservation analysis of *PAX9* amino acid sequences in six species. (c) WebLogo analysis of *PAX9* amino acid sequences in six species. (d) Structural modeling of the wild-type *PAX9* protein (the amino acids encoded by the 113Afs are shown in red). (e) Structural modeling of the PAX9 p.D113Afs*9 variant (the changed amino acids are shown in orange)

Multi-species conservation analysis showed that amino acids 113 and 337 were highly conserved in protein sequences of normal human, cattle, chicken, dog, house mouse, and rhesus monkey ( Fig. 3b ). In the WebLogo diagram, the overall height of the stack indicates the sequence retention at this position, whereas the height of symbols within the stack indicates the relative frequency of each amino acid or nucleic acid at this position. WebLogo analysis also showed that amino acids 113 and 337 were highly conserved ( Figure 3c ).

The homology modeling analysis of the *PAX9* protein showed that p.D113Afs*9 is a frameshift variant at the linker of α5 and α6 in the paired domain (PD), which leads to change in amino acid 113 from aspartic to alanine acid, and termination of translation at position 121. Structural modeling showed that the p.D113AFs*9 variant changed the conformation of the PD domain ( Figure 3 d-e ).

### *PAX9* genotype-phenotype analysis

**Figure 4:**
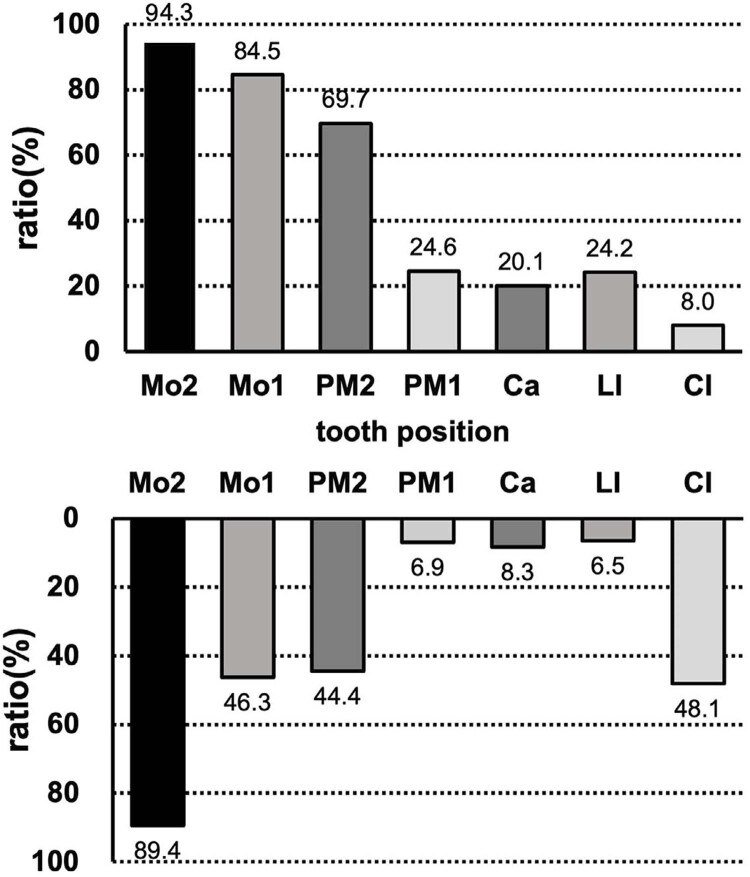
Phenotype composition of the 160 patients reviewed

**Figure 5:**
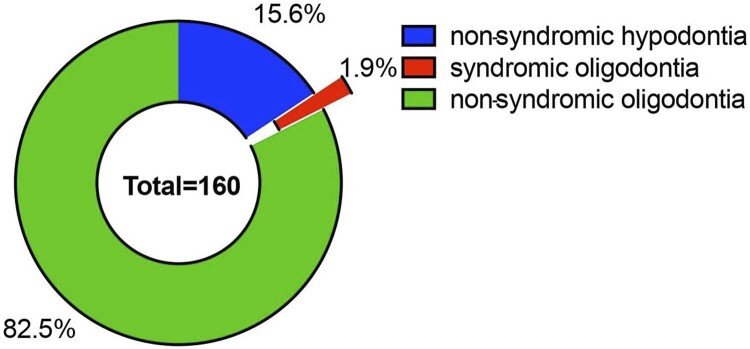
Permanent tooth loss rate of the upper and lower jaws of NSO patients with PAX9 variants (n=132)

We summarized 67 *PAX9* variant sites (160 patients) reported previously up to July 2022 ^10 , 12 - 51^and those identified in this study (Supplementary Table 1). We found that *PAX9* -related NSO accounted for 82.5% of the 160 patients. Of these, 15.6% had non-syndromic hypodontia and 1.9% had syndromic tooth agenesis ( Figure 4 ). Furthermore, evaluation of the characteristics of the *PAX9* -related NSO phenotype revealed that all types of permanent teeth can be missing, with a trend of left-right and up-down symmetry. In addition, the rate of maxillary tooth loss was slightly higher than the rate of mandibular tooth loss, with the exception of central incisors ( Figure 5 ). In descending order, the most likely teeth to be congenitally missing (>50%) were upper second molars (94.3%), lower second molars (89.4%), upper first molars (84.5%), and upper second premolars (69.7%) ( Figure 5 and Table 1).

## Discussion

The genetic heterogeneity of tooth agenesis is quite extensive, whereas non-syndromic oligodontia may have a certain genetic background and could aggregate in the family. According to Yu, et al.^52^(2019) more than 91.9% of non-syndromic tooth agenesis cases are caused by seven pathogenic genes ( *PAX9* ,^53 - 55^ *AXIN2* ,^56^ *EDA* ,^57^ *LRP6* ,^58 , 59^ *MSX1* ,^54 , 60 - 62^WNT10A,^59 , 63 - 65^and *WNT10B* ^12 , 66 , 67^). The non-syndromic oligodontia caused by *PAX9* variant is inherited in an autosomal dominant manner. In this study, we identified novel *PAX9* variants in a Chinese family with NSO.

In this Chinese family with NSO, the proband’s causative gene variant was inherited in an autosomal dominant pattern from the maternal pedigree. WES showed that the proband carried a compound heterozygous variant *PAX9* c.330_331insGT (p.D113Afs*9) with *PAX9* c.1010C>A (p.T337K) that co-segregates with congenitally missing teeth in the family; this was confirmed by Sanger sequencing. According to SIFT, Poly-Phen2, and MutationTaster, the two variants were predicted to be pathogenic. Analysis of multi-sequence species showed that the two variant sites were highly conserved. The three-dimensional structure reconstruction of the protein showed that the c.330_331insGT (p.D113Afs*9) variant caused a frameshift in the α5 and α6 linker regions of the PD, resulting in protein truncation ( Figure 3a ). PAX9 is an important transcription factor; the binding of PAX9 protein to target DNA is achieved through the N-terminal subdomain of the PD, whereas the C-terminal subdomain cooperates with the N-terminal subdomain to play a role in pathway regulation.^10^ Thus, this protein plays an important role in activating the odontogenic potential of the dental mesenchyme and, subsequently, in the process of tooth morphogenesis and formation.^6^

The *PAX9* c.1010C>A (p.T337K) variant is located at the carboxy terminus of exon 4, and results in a change of the corresponding position in the PAX9 protein from a polar uncharged threonine with a relatively small sidechain to a polar positively charged lysine with a longer sidechain. This variation affects interactions with surrounding amino acid residues and causes three-dimensional conformational changes. Although *PAX9* c.1010C>A (p.T337K) variant was found to be inherited among the patients in the pedigree, the truncated *PAX9* protein played a role in its pathogenicity. Thus, the pathogenic mechanism in this pedigree was protein truncation caused by the *PAX9* c.330_331insGT (p.D113Afs*9) variant.

To verify the more detailed features of *PAX9* -related tooth agenesis phenotypes, we reviewed cases from reported articles and a Chinese Han pedigree. We found bilateral symmetry is a characteristic of *PAX9* -related tooth agenesis in NSO, with the highest rates of loss in the mandibular and maxillary second molars, upper first molars, and upper second premolars, which is consistent with the findings reported by Liu, et al.^48^(2022). In addition, we found that upper teeth were more frequently missing than teeth in the same position of lower jaw, with the exception of central incisors. The lower central incisors were more often affected.

## Conclusions

We identified novel compound heterozygous variants c.330_331insGT (p.D113Afs*9) and c.1010C>A (p.T337K) in *PAX9* in a Chinese Han family with non-syndromic oligodontia. *PAX9* c.330_331insGT (p.D113Afs*9) leads to truncation of the *PAX9* protein in the PD domain and was predicted to be the pathogenic variant in this family. This expands the variant spectrum of *PAX9* and provides a basis for genetic diagnosis of this rare congenital anomaly.
